# 20 Does hip involvement affect foot and ankle in juvenile idiopathic arthritis?

**DOI:** 10.1093/rheumatology/keac496.016

**Published:** 2022-10-07

**Authors:** Moalla Myriam, Ferjani Hanene, Triki Wafa, Ben Nessib Dorra, Maatallah Kaouther, Kaffel Dhia, Hamdi Wafa

**Affiliations:** 1 Rheumatology Department, Kassab Orthopedics Institute, Ksar Saïd, Tunisia; 2 Faculty of Medicine of Tunis, University Tunis el Manar, Tunisia; 3 Research Unit UR17SP04, 2010, Ksar Said 2010, Tunis, Tunisia

**Keywords:** juvenile idiopathic arthritis, hip, foot, ankle

## Abstract

**Background:**

Although ankle and foot involvements are common in juvenile idiopathic arthritis (JIA), they are often neglected. Hip involvement, also common in JIA, may affect these joints by creating a chronic imbalance of the musculoskeletal system. However, no studies have been published on this subject.

**Objective:**

We aimed to describe ankle and foot impairment in a cohort of patients with JIA and to study the correlation between these impairments and the presence of hip arthritis.

**Methods:**

A monocentric cross-sectional study was conducted including JIA patients fulfilling the 2001 ILAR criteria. Patients with congenital malformation of the ankle or foot or with any other foot impairment due to a disease other than JIA were not included. Foot examination was performed on bare feet both in supine and standing position. We completed with an analysis of footprint with a podoscope and shoes examination. Patients were divided in two groups depending on the presence or the absence of hip arthritis on pelvis X-ray, hip ultrasound or hip magnetic resonance imaging.

**Results:**

We included 35 patients (M/F = 15/20). Hip arthritis was noted in 45.7%. Oligoarticular (43.8%) and enthesitis-related arthritis (25%) were the most frequent form of JIA. Functional complaints related to foot and ankle were reported in 34.3% of cases. The pain was the most frequent symptom (91%), mainly in the hindfoot and ankle (50%). Foot pain was more frequently encountered in the absence of hip arthritis (52%, *vs* 31.2% in presence of hip arthritis). Physical examination revealed limitation of the talocrural joint in 20% of cases and feet tenosynovitis in 14.3% of cases. Achille tendon enthesitis was found in 8.6% of patients. These abnormalities were more prevalent in the absence of hip arthritis. Half of the patients had hindfoot deviation dominated by hindfoot varus (22.9%). In the group with hip arthritis, a hallux valgus was found in 14.3%, a supraductus of the 2^nd^ toe, and claw toe in one case each. An anomaly of the footprint was noted in 28 patients, including 11 in the group with hip involvement: 7 cases of cavus foot and 4 cases of flat foot. There were no correlations between foot or ankle anomalies with hip impairment apart from an association of flat foot with the absence of coxitis, and a leg length discrepancy more important in the group with hip arthritis.

**Conclusions:**

Our study confirms the frequency of foot and ankle involvement as well as hip arthritis during JIA, hence the importance of their systematic screening even in asymptomatic children. Larger-scale studies would be necessary to evaluate with more precision the relation that there could be between hip and foot impairment.

